# Serum protein biomarkers for polymyalgia rheumatica: a case-control study

**DOI:** 10.3389/fimmu.2026.1948036

**Published:** 2026-09-09

**Authors:** Hiroshi Furukawa, Yuichi Hikichi, Kohei Kunieda, Shomi Oka, Takashi Higuchi, Shigeto Tohma, Kenji Itoh

**Affiliations:** 1 Department of Rheumatology, NHO Tokyo National Hospital, Kiyose, Japan; 2 Tuning Fork Bio Japan, MITSUI LINK-Lab SHINKIBA 2, Tokyo, Japan

**Keywords:** case-control study, polymyalgia rheumatica, protein biomarker, proteomic analyses, rheumatoid arthritis

## Abstract

**Objective:**

Polymyalgia rheumatica (PMR), a chronic inflammatory disease that affects older people, is characterized by musculoskeletal pain in the shoulder, neck, and pelvic girdle. It can be challenging to discriminate PMR from older age onset rheumatoid arthritis (RA). Systematic investigations of serum proteins have been conducted to identify new biomarkers. However, few proteomic analyses have been conducted for PMR. We analyzed serum protein profiles to identify biomarkers of PMR in the comparison between PMR patients before corticosteroid treatment [PMR Tx(-)] and healthy controls (HCs).

**Methods:**

Systematic proteome analyses of serum samples from 16 PMR Tx(-) and 18 HCs were performed and candidate proteins were selected with statistical/cluster and outlier analyses. Validation of the results was performed by the multiplex bead-based immunoassay of 10 candidate proteins in serum from 16 PMR Tx(-), 30 PMR after corticosteroid treatment [PMR Tx(+)], 26 RA, and 16 HCs.

**Results:**

C-C motif chemokine ligand (CCL)7, C-X-C motif chemokine ligand (CXCL)9, interleukin (IL)-6, matrix metalloproteinase (MMP)-8, interferon (IFN)-alpha, and MMP-3 levels were increased in PMR Tx(-) compared with HC. Area under the curve values of receiver operating characteristic curves of CXCL9, IL-6, and IL-1 receptor antagonist were 0.94, 0.97, and 0.94, respectively.

**Conclusions:**

Serum biomarker profiles could represent diagnostic biomarkers for PMR.

## Introduction

Polymyalgia rheumatica (PMR), a chronic inflammatory disease differentiated by musculoskeletal pain and morning stiffness of the shoulder, neck, and pelvic girdle (1) affects those older than 50 years. PMR in European (2) but not Japanese (3–5) populations are commonly complicated by giant cell arteritis. For European populations (6, 7), the prevalence of PMR is 600–800 per 100, 000 individuals older than 50 years, but only 250–300 in Japanese (5, 8). Whether PMR and malignancy are associated is controversial (9–12).

Rheumatoid arthritis (RA) is an inflammatory disease characterized by destruction of cartilage and bone. RA patients produced specific auto-antibodies including anti-citrullinated peptide antibodies and rheumatoid factor, though no specific autoantibodies have been detected in PMR patients. There are no specific clinical examinations for PMR; it can be difficult to discriminate PMR from older age onset RA. The etiology of PMR might be affected by genetic and environmental factors (13) and PMR is linked to autoimmune diseases and autoinflammatory disorders (2, 14). Acute phase reactants are markedly increased in autoinflammatory disorders and PMR but not increased in autoimmune diseases. However, increased serum levels of C-X-C motif chemokine ligand (CXCL)9, interleukin (IL)-6, CXCL10, and interferon (IFN)-gamma were reported in PMR patients (15–20).

Proteomic assays with modified single strand DNA (21) or antibody immunoassays and next generation sequencing (22, 23) have been developed and can be used to measure low levels of blood proteins with high specificity. Systematic investigations of blood proteins have been conducted to identify new biomarkers. However, few proteomic analyses have been conducted for PMR (17). As giant cell arteritis is a frequently associated disorder in European populations with PMR, the overlapping of biomarker proteins between PMR and giant cell arteritis is possible. However, because of the low overlap rates in Japanese populations, biomarker proteins used to detect PMR in Japanese could be used to discriminate PMR from giant cell arteritis. This study examined the blood protein profiles of PMR patients to identify biomarkers specific to PMR.

## Materials and methods

### Patients

Forty-six PMR patients, 26 RA patients (mean age ± standard deviation [SD]: 84.5 ± 2.4, male number: 3 [11.5%]), and 18 healthy controls (HCs, mean age ± SD: 68.4 ± 6.5, male number: 13 [72.2%]), were consecutively recruited from Tokyo National Hospital from September 2020 to September 2025. PMR patients fulfilled 2012 Provisional Classification Criteria for Polymyalgia Rheumatica or Bird’s Criteria for PMR (24, 25). RA patients satisfied the American College of Rheumatology criteria for RA or Rheumatoid Arthritis Classification Criteria (26, 27). Resistance to corticosteroid treatment was defined as the recurrence of clinical symptoms accompanying elevated levels of acute phase reactants whilst tapering corticosteroids at Tokyo National Hospital. Patients with corticosteroid resistance were administered disease modifying anti-rheumatic drugs and corticosteroids for the relapse of PMR. Whole blood was obtained from peripheral venous blood from the participants, serum samples were separated after centrifugation at 1500× g for 10 min, and stored at −80 °C before antibody detection.

The Research Ethics Committees of Tokyo National Hospital (190010) reviewed and approved this study. Informed consent (written) was given by study patients. The study was performed in accord with the tenets of the Helsinki Declaration.

### Proteome analyses of PMR patient serum samples

Proteome analysis of serum samples from 16 PMR patients before corticosteroid treatment [PMR Tx(-)] and 18 HCs used an antibody immunoassay and next generation sequencing (Olink Explore HT, Olink Proteomics, Uppsala, Sweden, https://olinkpanel.creative-proteomics.com/knowledge/pea-technology.html), as previously described (22, 23). Normalized protein expression (NPX) values were obtained to quantify the relative protein levels in the serum samples on a log_2_ scale (https://olinkpanel.creative-proteomics.com/knowledge/olink-data-analysis-process.html).

### Assessment of residual technical variability and quantile normalization

Although NPX values underwent Olink’s standard internal normalization, cumulative distribution plots of raw NPX values indicated marked between-sample differences in distributional shape, suggesting residual technical variability after standard processing (**Supplementary Figure 1**). To harmonize sample-wise distributions prior to downstream analyses, quantile normalization was applied to NPX-derived values using an in-house Tuning-Base™ platform (**Supplementary Figure 2**, showing raw data vs. quantile-normalization) (28–30). Unless otherwise stated, statistical differential analyses and one set of clustering analyses were performed on quantile-normalized values.

### Candidate protein selection with statistical/cluster selection

Because PMR is clinically heterogeneous, candidate protein selection was designed to capture (i) group-level differences between PMR and HC and (ii) proteins with a clear elevation in subsets of PMR patients. Candidate selection consisted of three complementary components—differential analysis, clustering selection, and outlier selection—and subsequent integration by Venn overlap. The overall workflow and overlaps are summarized in **Supplementary Figure 3**.

In the differential analysis for Criterion 1, Welch’s *t*-test was used to compare quantile-normalized protein values between PMR Tx(−) and HCs. Multiple testing correction was performed using false discovery rate q-values. Proteins were retained if they met Criterion 1: (a) q-value < 0.05 and (b) fold-change > 2. The fold-change was computed from quantile-normalized values derived from NPX. Because NPX values are on a log2 scale, the fold-change was calculated as $2^{\left(\mu_{PMR}-\mu_{HC}\right)}$, where μ denotes the group mean of quantile-normalized values.

For clustering selection using quantile-normalized values for Criterion 2, unsupervised hierarchical clustering was performed on the full proteome (5416 proteins) using Euclidean distance and Ward’s linkage. Based on the dendrogram structure, a “PMR cluster” was defined as a distinct branch enriched for PMR Tx(−) samples.

For clustering selection using raw NPX values with no quantile normalization for Criterion 3, hierarchical clustering was repeated using raw NPX values (i.e., NPX values without quantile normalization) with Euclidean distance and Ward’s linkage to assess the robustness of clustering structure to additional normalization. The PMR-enriched cluster was identified from the dendrogram, and proteins with higher relative abundance within this PMR cluster were extracted.

The three protein sets from Criteria 1–3 were integrated. Proteins supported by at least two of the three approaches (differential analysis, quantile-normalized clustering, raw-NPX clustering) were retained as the “statistical/cluster-selected” group.

### Candidate protein selection with outlier selection

To capture proteins with a clear elevation in a subset of PMR patients, two outlier filters were applied to quantile-normalized values. Thresholds were selected for exploratory purposes to avoid generating an excessively large candidate list (targeting ~100–200 hits), rather than being derived from a formal statistical model ^30^. A protein was considered associated with PMR in the outlier criteria when it met at least one of Criteria 4 or 5. Proteins were retained if they met Criterion 4: a protein was selected if at least one PMR Tx(−) sample exceeded (PMR mean + 1.5×SD), had a value >10, and the PMR mean was ≥1.1-fold of the HC mean, whereas all HC samples were <8. Proteins were retained if they met Criterion 5: a protein was selected if at least one PMR Tx(−) sample exceeded (PMR mean + 1.5×SD), had a value >8, and the PMR mean was ≥1.1-fold of the HC mean, whereas all HC samples were <6. Proteins supported by at least one of the two approaches (Criteria 4 or 5) were retained as the “outlier selected” group.

The final candidate set was derived from the intersection of the “statistical/cluster selected” and “outlier selected” groups.

### Measurement of proteins in PMR patient serum samples

The Bio-Plex suspension array system (Bio-Rad, Hercules, CA, USA), a multiplex bead-based immunoassay, was used to detect proteins in individual serum samples obtained from 46 PMR patients, 26 RA patients, and 16 HCs. Serum samples from 16 PMR patients were obtained from PMR Tx(-) and remaining samples from 30 PMR patients were obtained after corticosteroid treatment [PMR Tx(+)]. The Luminex Human Discovery Assay (R&D Systems) was used to detect C-C motif chemokine ligand 7 (CCL7), IFN-gamma, CXCL9, IL-6, matrix metalloproteinase (MMP)-3, MMP-8, CXCL10, IFN-α, IL-1 receptor antagonist (IL-1RA), and phospholipase A2 group VII (PLA2G7). Sera were diluted 1:5 for analyses.

### Statistical analysis

Differences related to the attributes of RA patients were analyzed by *t*-test or Fisher’s exact test with 2×2 contingency tables. Hierarchical cluster analyses achieved using the Euclidean distance and Ward’s method were used to generate heatmaps with dendrograms. Principal component analysis to discriminate among PMR Tx(-) and HCs was performed. A comparison of protein amounts in sera from PMR patients and HCs was performed using *t*-tests. Protein levels were compared between PMR Tx(-), PMR Tx(+), RA, and HCs on receiver operating characteristic (ROC) curves. Area under the curve (AUC) values for ROC curves with 95% confidence intervals (CIs) were determined. Estimations of optimized cut-off levels were determined based on the highest Youden index value. Eighty percent statistical power was attained when the AUC was ≥ 0.81, 0.81, 0.86, 0.77, and 0.78 for the comparison between [PMR Tx(-) and PMR Tx(+)], [PMR Tx(-) and RA], [PMR Tx(-) and HCs], [PMR and RA], and [PMR and HCs], respectively. Statistical significance was considered when *P* < 0.05.

## Results

### Characteristics of PMR patients

**Table 1** presents the clinical manifestations of PMR patients. No significant deference was detected between PMR Tx(-) and PMR Tx(+).

**Table 1:** Clinical manifestations of patients with PMR.

Phenotype	PMR Tx(-) (n=16)	PMR Tx(+) (n=30)	*P*-value
Mean age, years (SD)	77.7 (7.8)	78.8 (6.9)	0.6097
Male, n (%)	6 (37.5)	18 (60.0)	0.2167
Age at onset, years (SD)	77.4 (7.2)	77.9 (7.0)	0.8227
Corticosteroid dose at onset, prednisolone mg (SD)	15.0 (0.0)	16.2 (5.1)	0.3531
Complication of malignancy, n (%)	6 (37.5)	5 (16.7)	0.1535
Complication of giant cell arteritis, n (%)	0 (0.0)	0 (0.0)	1.0000
Corticosteroid resistance, n (%)	8 (50.0)	15 (48.3)	1.0000
RF, U/ml, (SD)	11 (10.4)	97 (456.6)	0.4531
ACPA, U/ml, (SD)	22 (83.3)	15 (78.4)	0.7915
ESR (mm/h)	82.4 (31.3)	74.3 (25.9)	0.3638
CRP (mg/dl)	7.9 (5.0)	8.2 (6.0)	0.8398
MMP3 (ng/ml)	273.5 (207.5)	219.5 (135.4)	0.3131

PMR: polymyalgia rheumatica, Tx(+): with corticosteroid treatment, Tx(-): without corticosteroid treatment, RF: rheumatoid factor, ACPA: anti-citrullinated peptide antibody, CRP: c-reactive protein, ESR: erythrocyte sedimentation rate, MMP-3: matrix metalloproteinase-3. Data are presented as the mean value or number of each group. Phenotype frequencies or standard deviations were shown in parenthesis. Differences were evaluated by *t*-test or Fisher’s exact test using 2×2 contingency tables.

### Proteome analyses

Proteome analyses of serum samples from 16 PMR Tx(-) and 18 HCs were conducted. NPX values were generated and are shown in **Supplementary Table 1**. Principal component analyses were performed with the protein biomarkers and successfully discriminated PMR Tx(-) from HCs (**Supplementary Figure 4**). Thus, serum protein profiles were skewed in PMR Tx(-) in the proteome analyses.

### Identification of candidate proteins associated with PMR

To characterize the protein markers associated with PMR, we compared the serum profiles of PMR Tx(-) against those of HCs using statistical/cluster and outlier analyses. First, we tried to identify proteins with group level differences in statistical/cluster analyses. Candidate proteins were identified in PMR Tx(-) relative to HC: 324 proteins met Criterion 1, 110 proteins met Criterion 2, and 122 met Criterion 3. The overlaps among these proteins are summarized in a Venn diagram (**Supplementary Figure S3A**). Full lists of proteins identified under each criterion are provided in **Supplementary Table S2**, and 145 proteins were selected according to at least two criteria in the statistical/cluster analyses.

Proteins with clear elevations in individual patients were also identified in PMR Tx(-) relative to HCs in the outlier analyses: 96 proteins met Criterion 4, and 88 proteins met Criterion 5. The overlaps among these proteins are summarized in a Venn diagram (**Supplementary Figure S3B**). Full lists of proteins identified under each criterion are provided in **Supplementary Table S3**, and 173 proteins were selected according to at least one criterion.

Finally, 25 proteins overlapped in the selections of identifying proteins with clear elevations in individual patients and group level statistical differences in statistical/cluster and outlier analyses (**Supplementary Figure S3C**, **Supplementary Table S4**).

### Serum protein biomarker profiles of PMR

**Figure 1:**
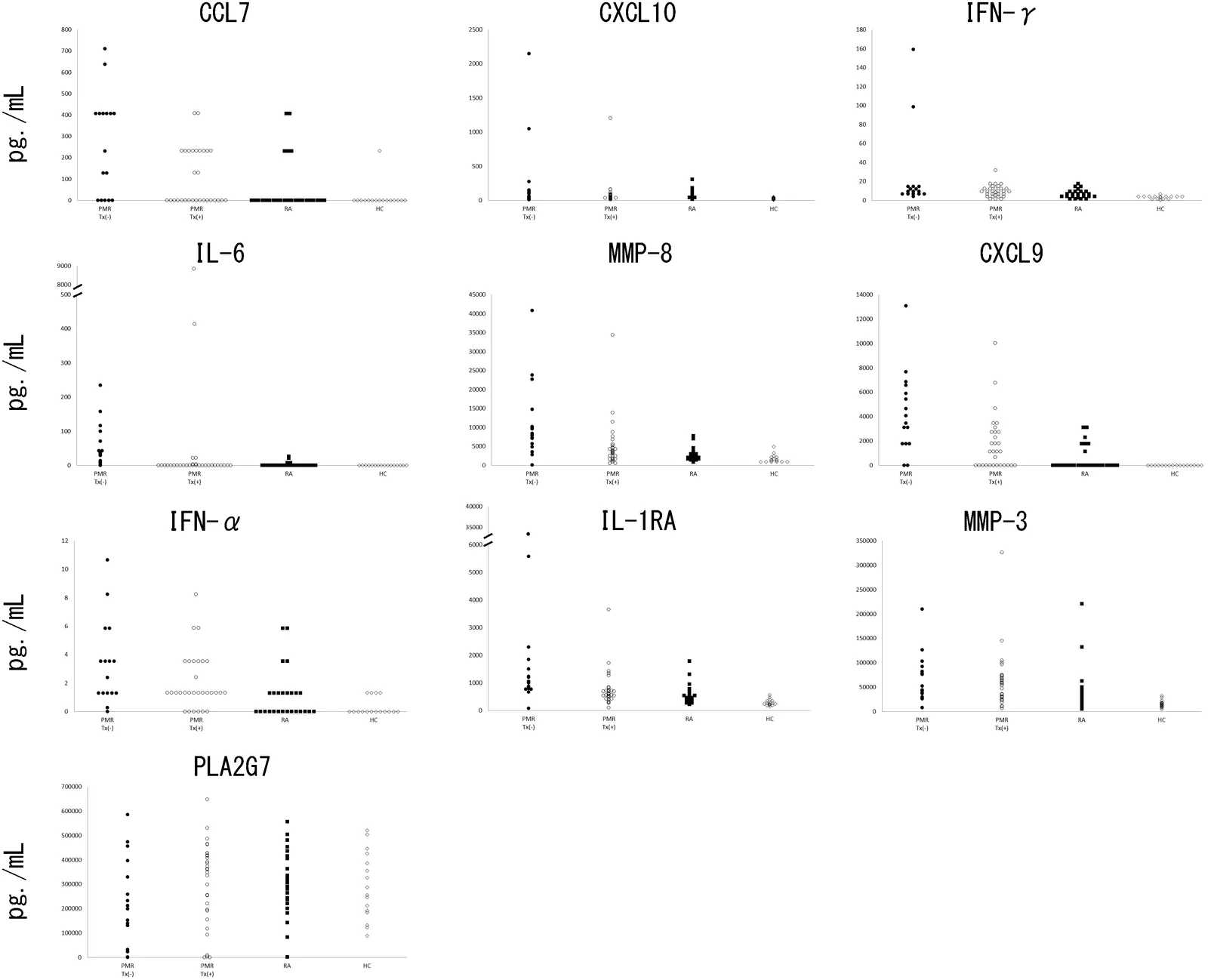
Validation of protein biomarker profiles of PMR and RA patients and HCs. Distribution of protein biomarkers are shown. Filled circles, empty circles, and filled squares, and empty diamonds represent PMR Tx(-), PMR Tx(+), RA, and HCs, respectively. PMR: polymyalgia rheumatica, RA: rheumatoid arthritis, HCs: healthy controls, Tx(+): with corticosteroid treatment, Tx(-): without corticosteroid treatment, CXCL: C-X-C motif chemokine ligand, CCL: C-C motif chemokine ligand, IL: interleukin, MMP: matrix metalloproteinase, IFN: interferon, IL-1RA: IL-1 receptor antagonist, PLA2G7: phospholipase A2 group VII.

Candidate protein markers were selected from the results of these proteome analyses. Ten candidate protein markers were selected from the 25 proteins in **Supplementary Table 4** and validated by multiplex bead-based immunoassays to detect proteins in individual sera from 16 PMR Tx(-), 30 PMR Tx(+), 26 RA, and 16 HC participants that included the patients used for the proteome analyses. The validation results are shown in **Table 2**. The levels of CCL7, IL-6, MMP-8, CXCL9, IFN-α, and MMP-3 were increased in PMR Tx(-) compared with HCs (**Figure 1**). The levels of MMP-8 and CXCL9 were also increased in PMR Tx(-) compared with PMR Tx(+). The levels of IFN-γ, IL-6, MMP-8, CXCL9, and IFN-α were increased in PMR Tx(-) compared with RA. The levels of CCL7, MMP-8, CXCL9, IFN-α, and MMP-3 were increased in PMR compared with RA or HCs. Thus, the levels of some serum protein biomarkers were skewed in PMR Tx(-).

**Table 2:** Protein concentrations in serum samples from patients with PMR or RA, or HCs.

	PMR Tx(-)	PMR Tx(+)	RA	HCs	PMR Tx(-) vs. PMR Tx(+)	PMR Tx(-) vs. RA	PMR Tx(-) vs. HC	PMR vs. RA	PMR vs. HC
					*P*	*P*	*P*	*P*	*P*
CCL7 (pg/mL)	267.72 (238.03)	105.15 (133.32)	58.05 (127.33)	14.46 (57.85)	0.0047	0.0006	0.0003	0.0159	0.0037
CXCL10 (pg/mL)	277.42 (557.97)	93.73 (213.25)	78.10 (65.97)	27.61 (12.71)	0.1149	0.0770	0.0835	0.2893	0.1734
IFN-γ (pg/mL)	24.51 (42.36)	9.74 (6.25)	6.68 (4.36)	3.32 (1.59)	0.0653	0.0381	0.0547	0.1156	0.0819
IL-6 (pg/mL)	58.27 (64.79)	310.46 (1614.94)	3.80 (7.87)	0.00 (0.00)	0.5377	0.0001	0.0011	0.3958	0.4989
MMP-8 (pg/mL)	11254.77 (10202.92)	5038.02 (6347.32)	2955.26 (1846.51)	1696.53 (1094.07)	0.0143	0.0002	0.0008	0.0129	0.0112
CXCL9 (pg/mL)	4332.84 (3305.66)	1750.69 (2291.95)	577.31 (1035.02)	0.00 (0.00)	0.0033	3.145X10^-6^	1.17X10^-5^	0.0009	0.0006
IFN-α (pg/mL)	3.38 (2.98)	2.07 (1.98)	1.13 (1.73)	0.33 (0.59)	0.0803	0.0034	0.0004	0.0119	0.0007
IL-1RA (pg/mL)	3368.70 (8097.19)	769.14 (649.56)	570.13 (344.82)	289.08 (109.94)	0.0845	0.0837	0.1387	0.2539	0.2624
MMP-3 (pg/mL)	67241.92 (50176.83)	61547.76 (59302.14)	37714.53 (45023.57)	15695.03 (7470.25)	0.7457	0.0551	0.0003	0.0477	0.0012
PLA2G7 (pg/mL)	226980.42 (180179.35)	301219.42 (171216.79)	310015.56 (133708.14)	292860.47 (136804.98)	0.1759	0.0950	0.2533	0.3873	0.7201

PMR: polymyalgia rheumatica, RA: rheumatoid arthritis, HCs: healthy controls, Tx(+): with corticosteroid treatment, Tx(-): without corticosteroid treatment, CXCL: C-X-C motif chemokine ligand, CCL: C-C motif chemokine ligand, IL: interleukin, MMP: matrix metalloproteinase, IFN: interferon, IL-1RA: IL-1 receptor antagonist, PLA2G7: phospholipase A2 group VII. Average value of each group was shown. Standard deviations are shown in parenthesis. Differences were evaluated by *t*-test.

### ROC curve analyses

**Figure 2:**
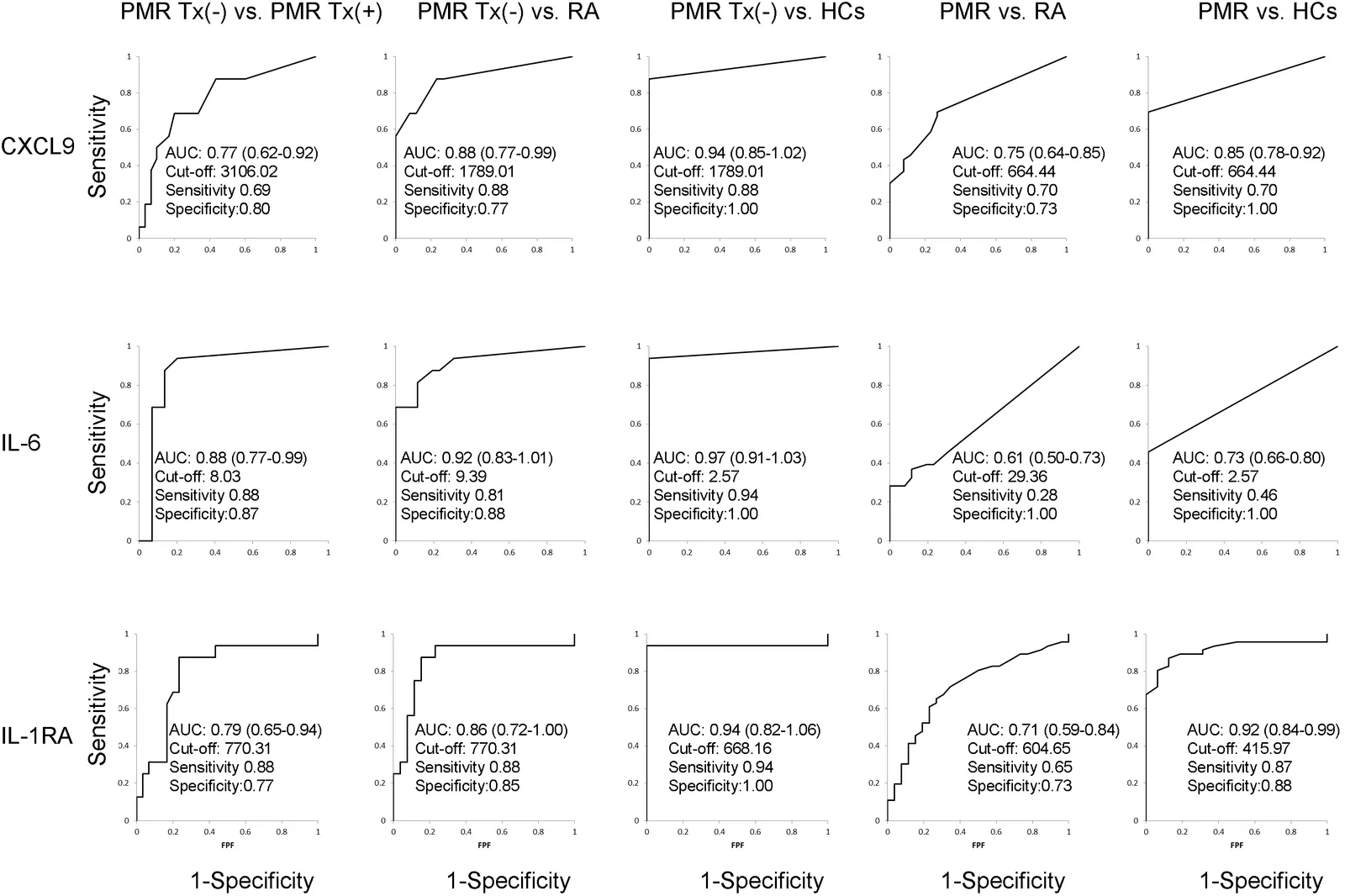
ROC curves using CXCL9, IL-6, and IL-1RA for comparisons between PMR Tx(-), PMR Tx(+), RA, and HCs. ROC curves for CXCL9, IL-6, and IL-1RA are shown. AUC values for ROC curves with 95% CIs were calculated. The optimized cut-off levels with specificities and sensitivities are described. ROC, receiver operating characteristic; AUC, area under the curve; CI, confidence interval; PMR, polymyalgia rheumatica; RA, rheumatoid arthritis; HCs, healthy controls; Tx(+), with corticosteroid treatment; Tx(-), without corticosteroid treatment; CXCL, C-X-C motif chemokine ligand; IL, interleukin; IL-1RA, IL-1 receptor antagonist.

The ROC curves for serum protein biomarkers were generated for comparisons between PMR Tx(-), PMR Tx(+), RA, and HCs (**Table 3**). The ROC curves of CXCL9, IL-6, and IL-1RA are shown in **Figure 2**. In the comparison between PMR Tx(-) and HCs, the AUC values of the ROC curves of CXCL9, IL-6, and IL-1RA were 0.94, 0.97, and 0.94, respectively. When compared between PMR Tx(-) and RA, those of CXCL9, IL-6, and IL-1RA were 0.88, 0.92, and 0.86, respectively. In the comparison between PMR and HCs, those of CXCL9, IL-6, and IL-1RA were 0.85, 0.73, and 0.92, respectively. When compared between PMR and RA, those of CXCL9, IL-6, and IL-1RA were 0.75, 0.61, and 0.71, respectively. Thus, CXCL9 or IL-1RA might be useful to discriminate PMR from other groups, and IL-6 might be useful to discriminate PMR Tx(-).

**Table 3:** AUC values of serum protein biomarkers for the discrimination of PMR.

	PMR Tx(-) vs. PMR Tx(+)	PMR Tx(-) vs. RA	PMR Tx(-) vs. HCs	PMR vs. RA	PMR vs. HCs
	AUC	AUC	AUC	AUC	AUC
CCL7	0.70 (0.53-0.87)	0.77 (0.62-0.91)	0.82 (0.70-0.95)	0.66 (0.55-0.77)	0.73 (0.64-0.83)
CXCL10	0.68 (0.51-0.86)	0.61 (0.42-0.79)	0.88 (0.75-1.00)	0.53 (0.39-0.66)	0.80 (0.69-0.91)
IFN-γ	0.59 (0.41-0.76)	0.76 (0.61-0.90)	0.97 (0.92-1.00)	0.69 (0.57-0.82)	0.90 (0.83-0.97)
IL-6	0.88 (0.77-0.99)	0.92 (0.83-1.00)	0.97 (0.91-1.00)	0.61 (0.50-0.73)	0.73 (0.66-0.80)
MMP-8	0.78 (0.62-0.93)	0.88 (0.75-1.00)	0.92 (0.80-1.00)	0.70 (0.58-0.82)	0.83 (0.73-0.94)
CXCL9	0.77 (0.62-0.92)	0.88 (0.77-0.99)	0.94 (0.85-1.00)	0.75 (0.64-0.85)	0.85 (0.78-0.92)
IFN-α	0.64 (0.47-0.80)	0.79 (0.65-0.92)	0.91 (0.81-1.00)	0.72 (0.59-0.84)	0.85 (0.76-0.94)
IL-1RA	0.79 (0.65-0.94)	0.86 (0.72-1.00)	0.94 (0.82-1.00)	0.71 (0.59-0.84)	0.92 (0.84-0.99)
MMP-3	0.57 (0.39-0.75)	0.77 (0.62-0.92)	0.93 (0.81-1.00)	0.73 (0.60-0.85)	0.90 (0.82-0.98)
PLA2G7	0.62 (0.44-0.80)	0.67 (0.48-0.86)	0.61 (0.41-0.81)	0.56 (0.42-0.69)	0.52 (0.36-0.67)

PMR, polymyalgia rheumatica; RA, rheumatoid arthritis; HCs, healthy controls; Tx(+), with corticosteroid treatment; Tx(-), without corticosteroid treatment; AUC, area under the curve; ROC, receiver operating characteristic; CXCL, C-X-C motif chemokine ligand; CCL, C-C motif chemokine ligand; IL, interleukin; MMP, matrix metalloproteinase; IFN, interferon; IL-1RA, IL-1 receptor antagonist; PLA2G7, phospholipase A2 group VII. An AUC value for each ROC curve was shown; 95% CIs are shown in parenthesis. Deviations from 0.5 were evaluated by chi-square tests.

## Discussion

The candidate protein approach in this study evaluated several protein markers for PMR (15–20), because few proteomic studies have investigated the roles of cytokines or chemokines in PMR (17). These studies suggested that CXCL10, CXCL9, IL-6, and MMP-3 might be candidate protein markers for PMR. Levels of IL-1RA were increased in PMR (31, 32) and polymorphisms in the *IL1RN* gene, which encodes the IL-1RA protein, were associated with PMR (33). In the present study, CXCL9, IL-6, and IL-1RA were found to be diagnostic biomarkers for PMR. These candidate protein markers were shared with the results of previous studies and the results would be robust.

Olink platforms were enriched for immune- and inflammation-related proteins; therefore, upregulated immune- and inflammation-related proteins would be selected. The 10 selected proteins in this study included immune- and inflammation-related proteins. A validation study using a Luminex assay showed CXCL9, IL-6, and IL-1RA were diagnostic biomarkers for PMR. CXCL9 associates with CXCR3 to regulate macrophages, cytotoxic lymphocytes, and natural killer cells (34). Targeting chemokine pathways may represent potential therapeutic strategies for PMR (35). IL-6 interacts with the IL-6 receptor (IL-6R) to initiate a signal transduction cascade through Janus kinases (JAKs). Thus, IL-6R and JAKs are potential targets for PMR treatment (18). Although IL-6 levels in patients in the PMR Tx(+) group were lower than those in the PMR Tx(-) group, levels in two patients in the PMR Tx(+) group were extremely high. This marked inter individual heterogeneity of PMR suggested a different pathogenesis among PMR patients and that disease in some PMR patients might be influenced by deleterious rare variants of autoinflammatory disorder-related genes (5). IL-1RA is an antagonist of the IL-1 receptor, preventing the signal transduction of IL-1 to cells. Thus, a recombinant IL-1RA, anakinra, might be useful for the treatment of PMR although further studies are required (36). This association with IL-1RA revealed features of autoinflammatory disorders might be present in PMR (2, 14). Some of these protein biomarkers might be used for estimation of corticosteroid resistance and it would be useful to select patients to be treated with disease modifying anti-rheumatic drugs. Implication of these protein biomarkers in the pathogenesis of PMR was suggested; however, the precise roles of these protein biomarkers are still unknown.

## Conclusion

In the present study, statistical/cluster and outlier analyses successfully selected 25 candidate protein markers from the results of proteomic analyses. In the validation analyses, CXCL9, IL-6, and IL-1RA were found to be diagnostic biomarkers for PMR. These results suggested different pathogenic pathways might be involved in PMR and RA. A limitation of this study was its small sample size. Age and sex could be possible confounding factors in the process of generating protein biomarkers, and AUC values were unadjusted exploratory estimate. PMR is frequently complicated with giant cell arteritis in European populations, but not in Japanese populations, suggesting a different pathogenesis of PMR between European and Japanese populations. This study was conducted in a Japanese population and PMR patients with giant cell arteritis was not included. These biomarkers should be verified in large-scale studies of different ethnic populations.

## Data Availability

The original contributions presented in this study are included in the article/**Supplementary Material**, further inquiries can be directed to the corresponding author.
