# Supplementary material for: Serum protein biomarkers for polymyalgia rheumatica: a case-control study
Source: Front Immunol. 2026 Sep 9;17:1948036. doi: 10.3389/fimmu.2026.1948036 (PMC13597238; doi:10.3389/fimmu.2026.1948036)
Supplement: Supplementary file 1 [file DataSheet1.pdf]

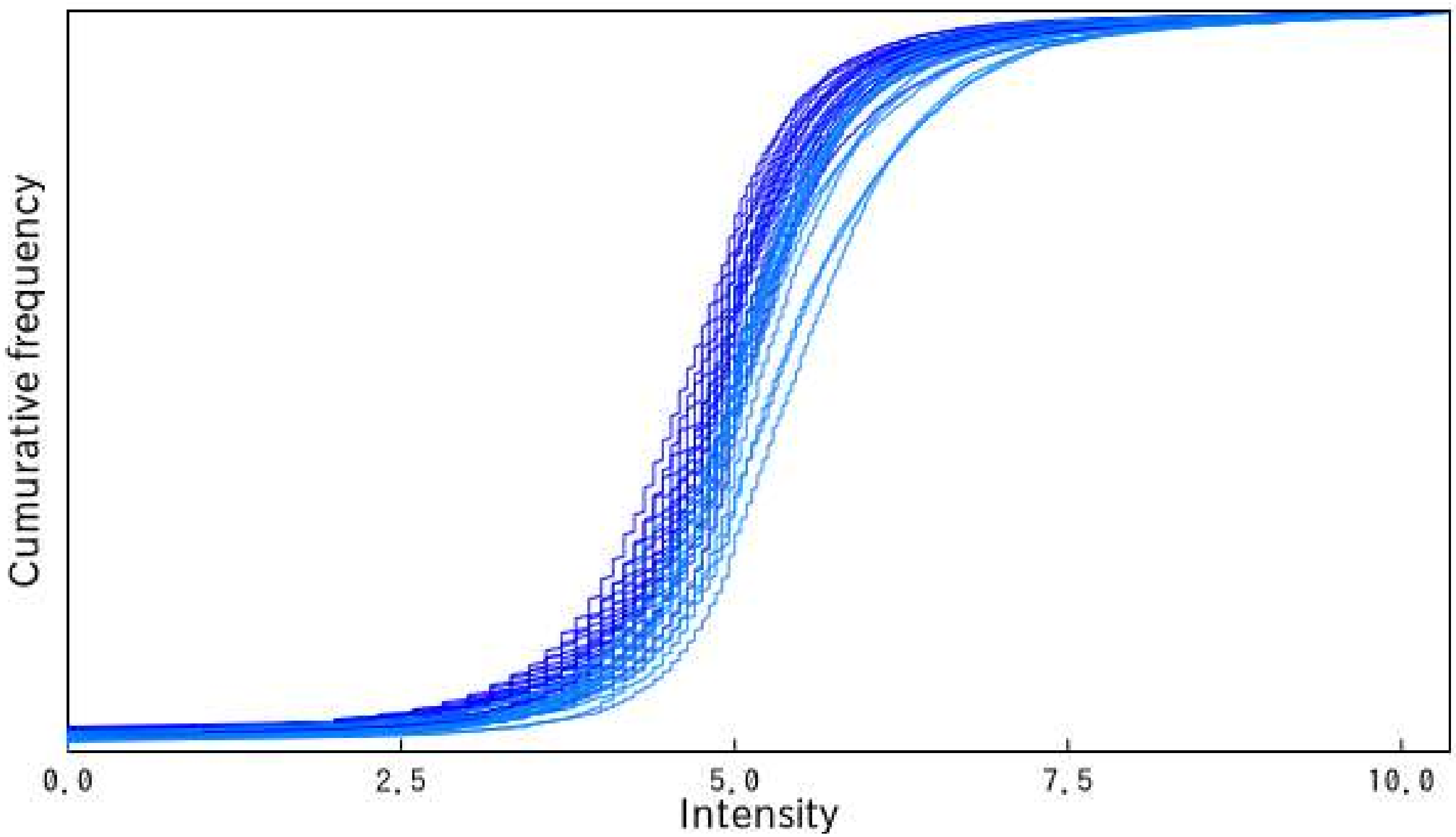

Supplementary Figure S1. Cumulative distribution plots of raw NPX values from all samples prior to normalization. The curves show that the NPX distributions differed markedly between samples, indicating residual technical variability after standard Olink processing. These plots were generated using the Tuning-Base™ software and motivated the application of quantile normalization to harmonize sample wise distributions before downstream analysis. NPX: normalized protein expression.
