# Supplementary material for: Serum protein biomarkers for polymyalgia rheumatica: a case-control study
Source: Front Immunol. 2026 Sep 9;17:1948036. doi: 10.3389/fimmu.2026.1948036 (PMC13597238; doi:10.3389/fimmu.2026.1948036)
Supplement: Supplementary file 2 [file DataSheet2.pdf]

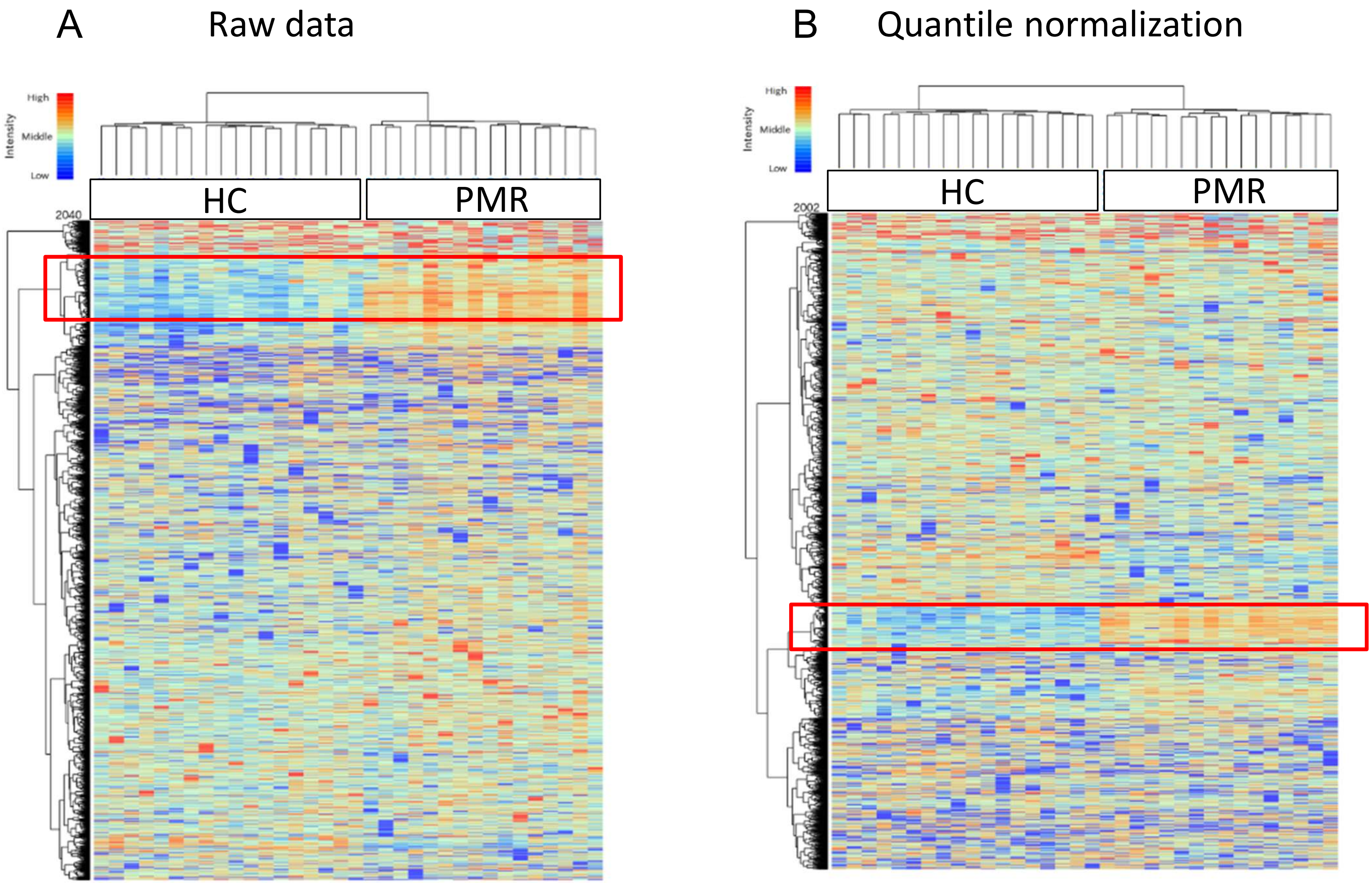

Supplementary Figure S2. Hierarchical clustering using protein levels in PMR and HCs. Heat map and dendrograms based on all 5416 protein levels in PMR and HCs. Clustering was performed from raw data (A, Criterion 3) or quantile-normalized NPX (log_2 scale) values (B, Criterion 2) using Euclidean distance and Ward's minimum variance linkage. Columns represent samples and rows represent proteins; the proteins upregulated in PMR were selected in red rectangles. PMR: polymyalgia rheumatica, HCs: healthy controls, NPX: normalized protein expression.
