# Supplementary material for: Serum protein biomarkers for polymyalgia rheumatica: a case-control study
Source: Front Immunol. 2026 Sep 9;17:1948036. doi: 10.3389/fimmu.2026.1948036 (PMC13597238; doi:10.3389/fimmu.2026.1948036)
Supplement: Supplementary file 3 [file DataSheet3.pdf]

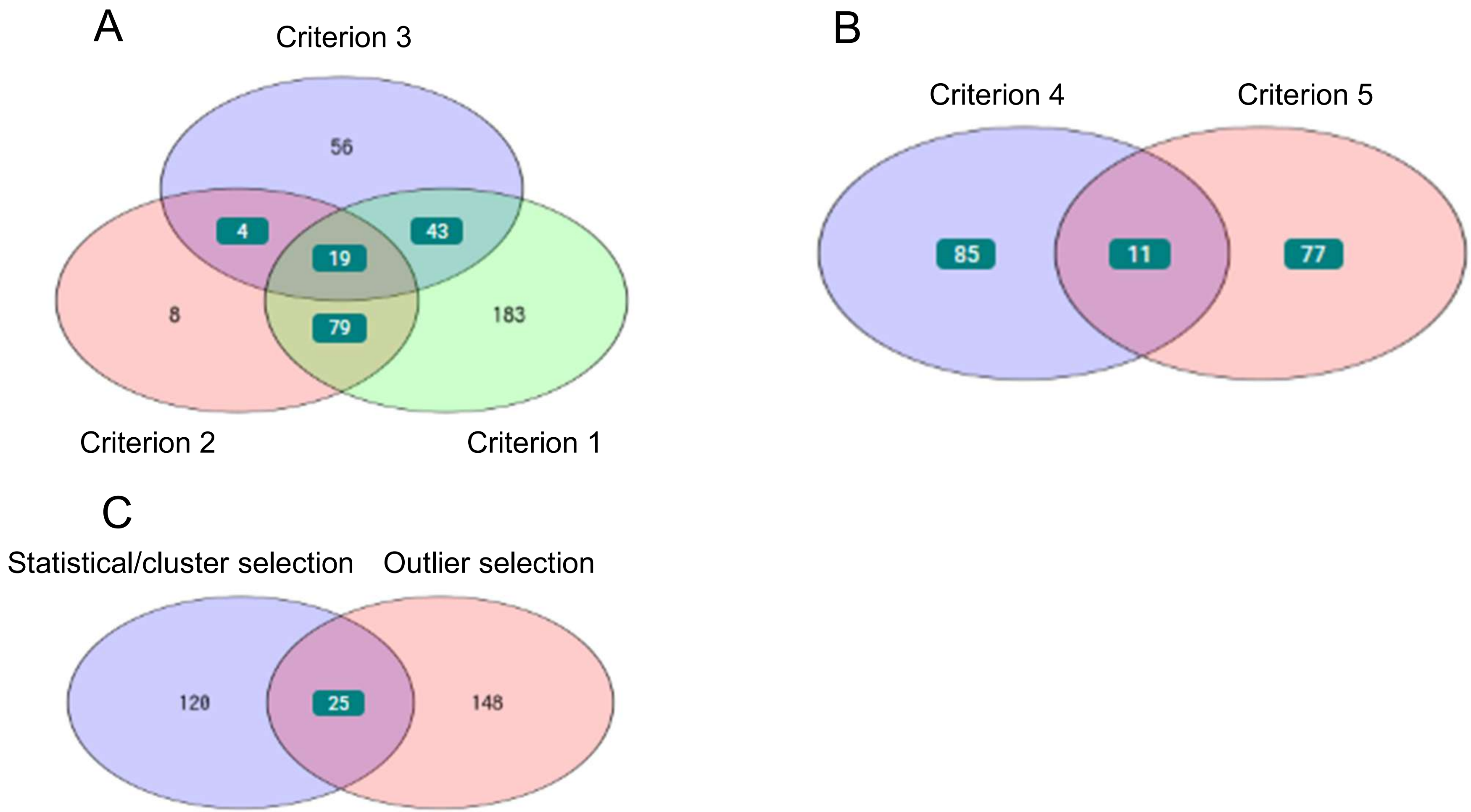

Supplementary Figure S3. Protein biomarker candidates of PMR patients compared with HCs. Protein biomarker candidate numbers for PMR with statistical/cluster selection (A), outlier selection (B), or both (C). PMR: polymyalgia rheumatica, HCs: healthy controls.
