# Supplementary material for: Serum protein biomarkers for polymyalgia rheumatica: a case-control study
Source: Front Immunol. 2026 Sep 9;17:1948036. doi: 10.3389/fimmu.2026.1948036 (PMC13597238; doi:10.3389/fimmu.2026.1948036)
Supplement: Supplementary file 4 [file DataSheet4.pdf]

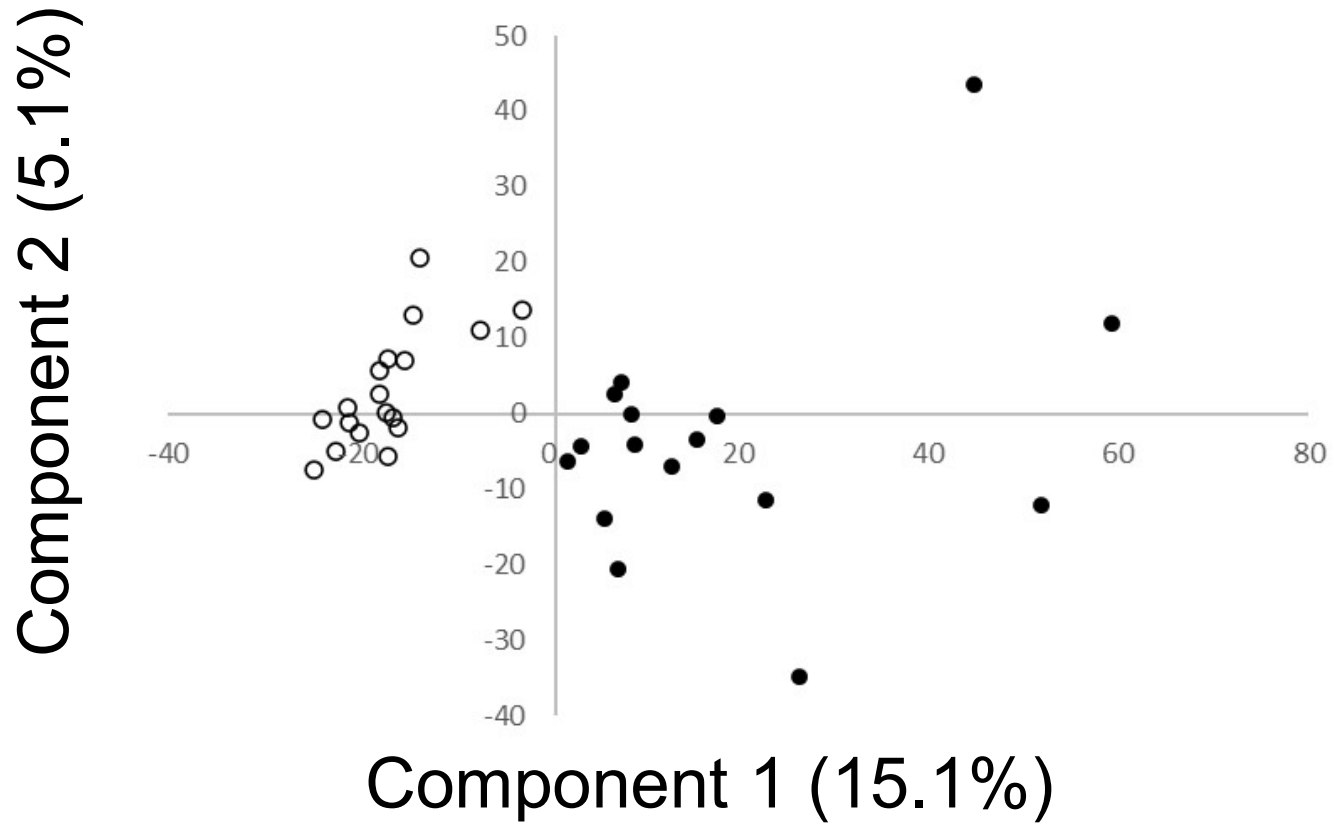

Supplementary Figure S4. Results of principal component analyses of protein biomarkers from the PMR or HC participants. Scores plots for principal component analyses of protein biomarkers. Filled circles and empty circles represent PMR and HCs, respectively. PMR: polymyalgia rheumatica, HCs: healthy controls.
